# Efficacy and safety of pembrolizumab on cervical cancer: A systematic review and single-arm meta-analysis

**DOI:** 10.3389/fonc.2022.910486

**Published:** 2022-08-10

**Authors:** Lin Qi, Ning Li, Aimin Lin, Xiuli Wang, Jianglin Cong

**Affiliations:** Department of Gynecology and Obstetrics, Yantai Yuhuangding Hospital Affiliated to Qingdao University, Yantai, China

**Keywords:** pembrolizumab, cervical cancer, meta-analysis, single-arm, systematic review

## Abstract

**Background:**

According to current research, the objective response rate and overall survival of pembrolizumab in the treatment of several types of solid tumors have been significantly improved. Some high-quality clinical trials have studied the effect of applying pembrolizumab in treating cervical cancer. Multiple clinical trials have been conducted, and some of them have shown good results as expected. Therefore, we performed this meta-analysis on existing studies to reveal the efficacy and safety of pembrolizumab in treating cervical cancer.

**Methods:**

PubMed, Embase, Cochrane Library and Web of Science were searched for literatures published until October 31, 2021. Outcomes included complete response (CR), partial response (PR), stable disease (SD), disease progression (PD), objective response rate (ORR), disease control rate (DCR), overall survival (OS), progression-free survival (PFS), the best time to response (TTR), death rate, adverse events (AE).

**Results:**

A total of 7 studies with 727 patients were included. The results were as follows: CR (0.027, 95%CI: 0.008-0.053), PR (0.104, 95% CI: 0.074-0.145), SD (0.190, 95% CI: 0.149-0.240), PD (0.541, 95% CI: 0.421-0.661). ORR was 0.155 (95% CI: 0.098-0.236) and DCR was 0.331 (95% CI: 0.277-0.385). OS was 10.23 months (95% CI: 8.96-11.50) and PFS was 4.27 months (95% CI: 1.57-6.96). TTR was 2.10 months (95%CI: 1.69-2.51). The 1-year death rate was 0.388 (95% CI: 0.230-0.574). Main adverse events included abnormal liver function, hypothyroidism, neutropenia, anemia, decreased appetite, fatigue, fever, etc. The total incidence of the adverse events of grade 3 and above was 0.212 (95% CI: 0.065-0.509).

**Conclusions:**

Pembrolizumab provides significant benefits in response rate and survival for cervical cancer patients. The results from recent high-quality clinical trials are expected to validate these findings.

**Systematic Review Registration:**

https://www.crd.york.ac.uk/prospero/, identifier CRD42021291723.

## 1 Introduction

Cervical cancer is the most common malignancy in female reproductive system. It is the fourth most common cancer in women worldwide and the fourth leading cause of cancer-related death (1). With the popularization of HPV vaccine and applying systemic screening of disease in recent years, the incidence of cervical cancer has decreased significantly in developed countries (2–4), but the incidence and mortality rate in developing countries still remained high (5, 6). According to the statistics by World Health Organization, in 2020, an estimated 604,127 women will be diagnosed with cervical cancer globally and 341,831 women will die from the disease, with approximately 90% of the cases occurring in low- and middle-income countries (7). The treatment of cervical cancer is mainly determined by the disease stage at diagnosis, and the commonly used methods include surgical resection, radiotherapy and chemotherapy (8). Among the cervical cancer patients at early stage without evidence of lymph node metastasis, approximately 11-22% of them relapse after receiving primary standard treatment. In patients with lymph node metastasis and/or disease progression at local lesion, the recurrence rate is as high as 28-64% (9, 10). Compared with the cervical cancer patients at an early stage, patients with recurrent/metastatic cervical cancer have a poor prognosis, which is an important cause of death. In addition, the treatment approaches are quite limited. The effects of traditional chemotherapy are unsatisfactory, and there are various complications related to surgery and radiotherapy. Thus, choosing proper therapeutic strategies for patients with recurrent/metastatic cervical cancer remains challenging and is a tough issue in the treatment of gynecological tumors (11, 12). Therefore, improving the efficacy of cervical cancer treatment has been a major medical challenge worldwide.

In 1992, PD-1 was first discovered in mice (13) as a member of the CD28 superfamily, and PD-L1 (also known as CD274 or B7-H1) was its ligand (14). PD-L1 is expressed by tumor cells and can bind with PD-1 to inhibit the activation of T cell and cytokine production. PD-1/PD-L1 antibody blocks the pathway by binding to PD-1/PD-L1, which contributes to the growth and proliferation of T cells, reduces the apoptosis of T cell, activates the attack and killing abilities of T cell, and restores the sensitivity of the immune response to enhance antitumor activity (15, 16). At present, there are a large number of clinical trials evaluating the efficacy and safety of PD-1/PD-L1 immune checkpoint inhibitors (ICIs) in patients with different types of tumors (17). It has been found that PD-L1 expression was increased in HPV-induced cervical cancer, suggesting that PD-1 may be an effective therapeutic target for cervical cancer patients (18). Among them, pembrolizumab is a highly selective human monoclonal antibody that can block the interaction between PD-1 and PD-L1, thereby promoting the process of killing tumor cells by immune system (19). According to statistics, since pembrolizumab was first approved to be used in the treatment of advanced melanoma in September 2014, at least 500 clinical studies have been conducted on 20 solid tumors and hematological malignancies (20). In June 2018, FDA approved pembrolizumab for recurrent/metastatic cervical cancer patients who had tumors expressing PD-L1 after chemotherapy or had progressed disease. To date, approximately seven clinical trials or case series have reported the final or mid-term results of studies on the efficacy of pembrolizumab in patients with advanced cervical cancer (21–27), and three other trials are in progress (NCT04221945, NCT05007106, EUCTR2020 -000172-38-FR). There is still a lack of evidence supported by evidence-based medicine for using pembrolizumab in patients with advanced cervical cancer. Therefore, we performed this systematic review and single-arm meta-analysis to assess the efficacy and safety of pembrolizumab in patients with advanced cervical cancer.

## 2 Materials and methods

This meta-analysis strictly abided by the PRISMA statement and was registered on PROSPERO (registration number CRD42021291723).

### 2.1 Search strategy

We searched PubMed, Embase, Cochrane Library and Web of Science databases for eligible studies published before 31 October 2021. Subject terms and free terms were used in the search. The subject terms used in PubMed were Uterine Cervical Neoplasms [Mesh], and pembrolizumab [Mesh]. The detailed search strategy is shown in **Table 1**.

**Table 1:** Search strategy in PubMed.

Search number	Query	Results
#1	“Uterine Cervical Neoplasms”[Mesh]	79,144
#2	((((((((((((((((((((((((Uterine Cervical Neoplasms[Title/Abstract])) OR (Cervical Neoplasm, Uterine[Title/Abstract])) OR (Cervical Neoplasms, Uterine[Title/Abstract])) OR (Neoplasm, Uterine Cervical[Title/Abstract])) OR (Neoplasms, Uterine Cervical[Title/Abstract])) OR (Uterine Cervical Neoplasm[Title/Abstract])) OR (Neoplasms, Cervical[Title/Abstract])) OR (Cervical Neoplasms[Title/Abstract])) OR (Cervical Neoplasm[Title/Abstract])) OR (Neoplasm, Cervical[Title/Abstract])) OR (Neoplasms, Cervix [Title/Abstract])) OR (Cervix Neoplasms[Title/Abstract])) OR (Cervix Neoplasm[Title/Abstract])) OR (Neoplasm, Cervix[Title/Abstract])) OR (Cancer of the Uterine Cervix[Title/Abstract])) OR (Cancer of the Cervix[Title/Abstract])) OR (Cervical Cancer[Title/Abstract])) OR (Uterine Cervical Cancer[Title/Abstract])) OR (Cancer, Uterine Cervical[Title/Abstract])) OR (Cancers, Uterine Cervical[Title/Abstract])) OR (Cervical Cancer, Uterine[Title/Abstract])) OR (Cervical Cancers, Uterine[Title/Abstract]])) OR (Uterine Cervical Cancers[Title/Abstract])) OR (Cancer of Cervix[Title/Abstract])) OR (Cervix Cancer[Title/Abstract])) OR (Cancer, Cervix[Title/Abstract])) OR (Cancers, Cervix[Title/Abstract])	79,729
#3	#1 OR #2	103,648
#4	“pembrolizumab” [Supplementary Concept]	2,745
#5	((((pembrolizumab[Title/Abstract]) OR (SCH-900475[Title/Abstract])) OR (Keytruda[Title/Abstract])) OR (MK-3475[Title/Abstract])) OR (lambrolizumab[Title]/Abstract])	5,612
#6	#4 OR #5	6,269
#7	#3 AND #7	62

### 2.2 Inclusion and exclusion criteria

This study was a meta-analysis based on published data, and the inclusion criteria were as follows (1): the literature which research subject was on human cervical cancer patients (2); single-arm study or RCT in which the intervention method was pembrolizumab treatment (3); English literature. Exclusion criteria (1): in-vitro experiments, reviews, abstracts, letters, pathological studies, etc. (2) literature in other languages (3); original literature unavailable. If authors published several studies using the same data, the most recent or comprehensive ones were included.

### 2.3 Literature screening and data extraction

Two investigators independently screened the literature according to the inclusion and exclusion criteria to determine the final included studies, and then independently extracted information. Before information extraction, a standard spreadsheet for information extraction was created and the extracted data included basic information (article title, first author, publication year, national clinical trial (NCT) registration number, country, study type, intervention method, sample size, age, disease stage and type) and outcome indicators [complete response (CR), partial response (PR), stable disease (SD), disease progression (PD), objective response rate (ORR), disease control rate (DCR), overall survival (OS), progression-free survival (PFS), the best time to response (TTR), death rate, adverse events (AE)].

Two researchers independently screened the literature, extracted information and then cross-checked their work. If there was any dissent, a third researcher was consulted to make a determination.

### 2.4 Quality Evaluation

The quality of the included studies was assessed using the Methodological Evaluation Metrics for Non-Randomized Controlled Trials (MINORS) (28). MINORS includes 12 evaluation indicators and each one can be scored 0-2. The first 8 items are for studies without a control group and the maximum score is 16. The last 4 items and the first 8 items are for studies with a control group and the maximum score is 24. 0 means that the data is not reported. 1 means that the data is reported but without sufficient information. 2 means that the data is reported with sufficient information.

### 2.5 Statistical analysis

R4.04 software (R development Core Team, Vienna, http://www.R-project.org) with metafor, matrix and meta packages was used to conduct single-arm meta-analysis. I^2^ was used to measure the heterogeneity, and the Q test was used to test the significance of heterogeneity. When I^2^ ≥ 50%, heterogeneity was considered to be significant, and the random-effects model was used to pool the effect size. When I^2^ <50%, the fixed-effects model was used to pool effect size.

## 3 Results

### 3.1 Selection of literature

**Figure 1:**
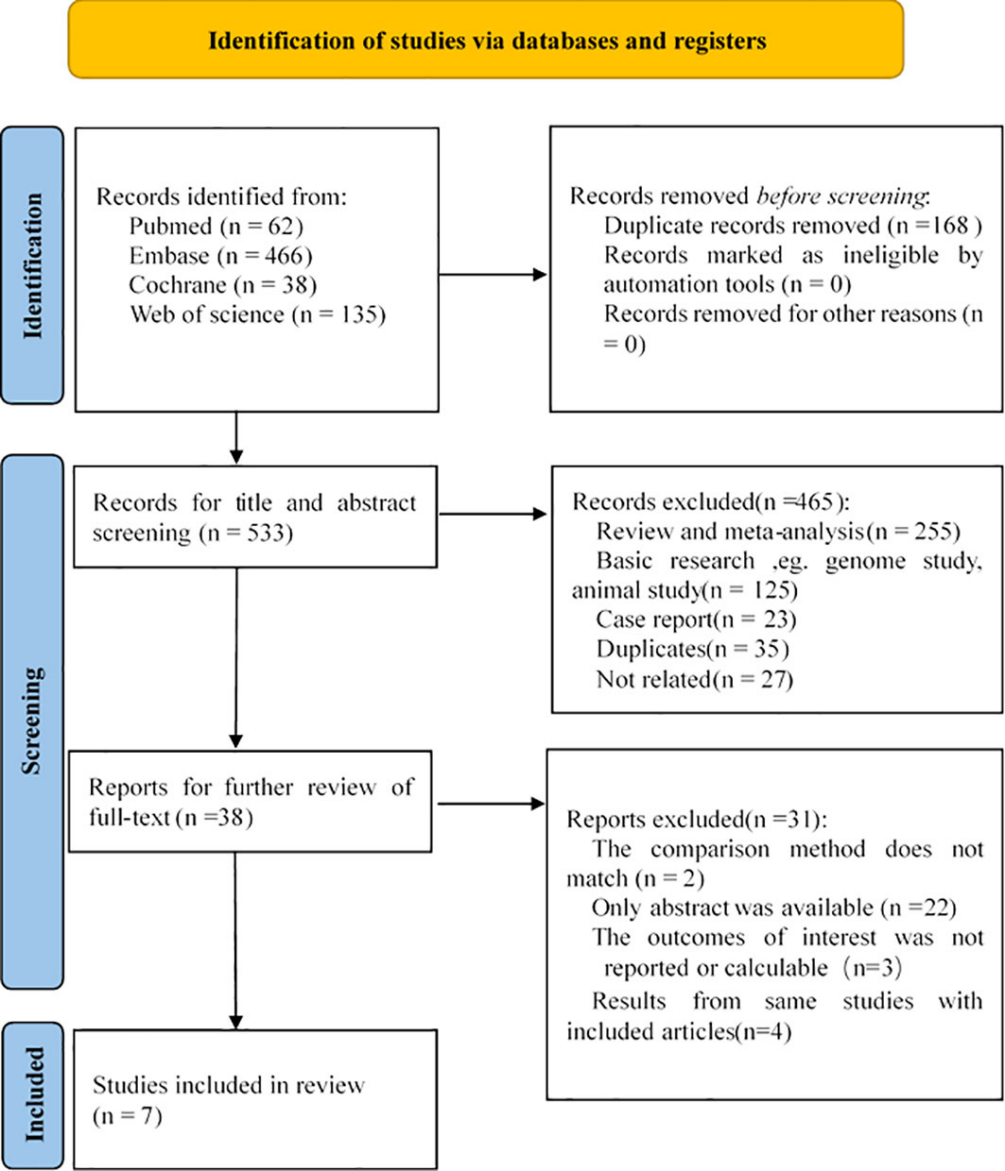
Flow chart of studies selection process.

After a thorough search, a total of 701 related studies were found, of which 62 studies were from PubMed, 466 studies from Embase, 38 studies from Cochrane, and 135 studies from WOS. By reading the titles, abstracts and full texts, meta-analyses, reviews, letters, conference abstracts, animal experiments, and case reports were excluded, and 7 studies were finally included in our research (21–27). The selection process is shown in **Figure 1**. The 7 selectively included articles were assessed and checked by two investigators. According to the MINORS scale, the included articles had a high level of methodological quality.

### 3.2 Basic characteristics of the included studies

Seven studies with a total of 727 patients were included, of which 1 study was phase III RCT, 4 phase II RCTs, 1 phase I RCT and 1 observational study. Seven studies were published within the past 5 years (2017–2021), which indicated that this study was innovative and the immune checkpoint inhibitors were developed rapidly in cervical cancer treatment. Six of the studies used monotherapy of pembrolizumab and one used combination therapy. The main characteristics, treatment strategies and quality evaluation of the included articles are shown in the **Table 2**.

**Table 2:** Characteristic of included studies.

First author	Year of publication	study design	Registration Number	Number of cases	Case country	tumor stage	score	literature
Hyun Cheol Chung	2019	international, open-label, multicohort phase II study	NCT02628067	98	17countries	II-1, IIIB-4I, VA-1, IVB-92	16	(21)
Nicoletta Colombo	2021	double-blind, phase 3 trial RCT	NCT03635567	308	19countries	I-67, II-85, III-5, IIIA-4, IIIB-46, IVA-7, IVB-94	24	(22)
Zachary Alholm	2021	observational study, retrospective cohort study		19	USA	0-IIA-15, IIB-IVA-61, IVB-39, Not documented-15	13	(23)
Kathryn M Miller	2021	retrospective cohort study		14	USA	IB-3, II-2, III-5, IV-4	12	(24)
Jin Won Youn	2020	open-label, single-arm, phase 2 trial	NCT03444376	36	South Korea		16	(26)
Min Chul Choi	2020	Multi-Center Retrospective Study		31	South Korea	I-31, II-34, III-22, IV-27, unknown-3	14	(25)
Jean-Sebastien Frenel	2017	multicenter, phase Ib, single-arm	NCT02054806	24		MX-1, M0-6, M1-15, Unknown-2	16	(27)

### 3.3 Results of meta-analysis

#### 3.3.1 Response rate (%) of CR, PR, SD and PD

**Figure 2:**
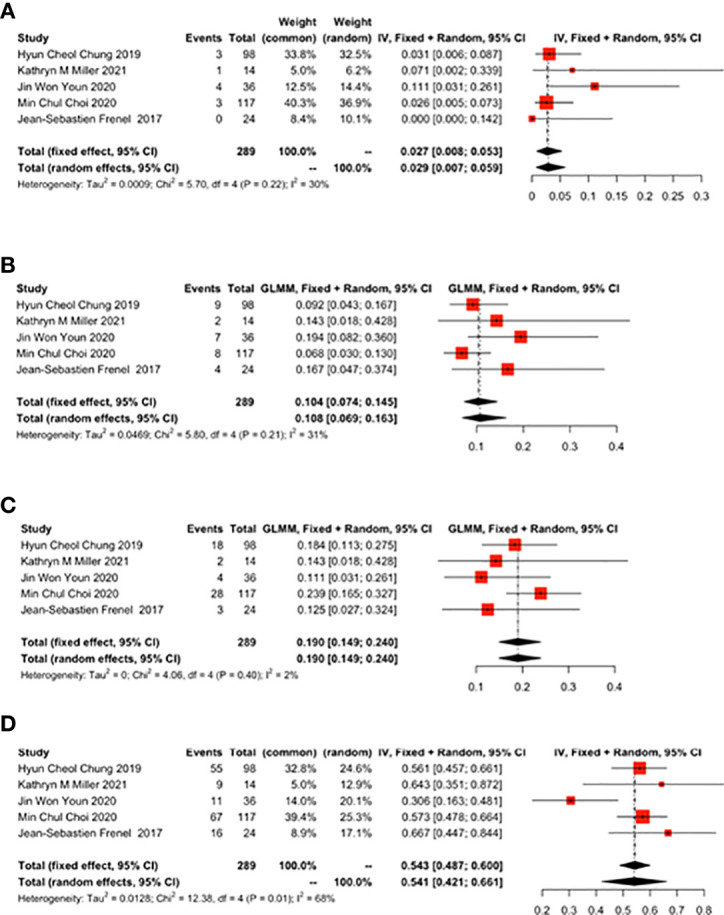
Complete response (CR), partial response (PR), stable disease (SD), disease progression (PD) for patients with cervical cancer receiving pembrolizumab therapy. **(A)** CR, **(B)** PR, **(C)** SD, **(D)** PD.

Five studies reported the response rate (%) of CR, PR, SD and PD. For cervical cancer patients treated with pembrolizumab, 11 out of 289 patients achieved CR (0.027, 95%CI: 0.008-0.053) (**Figure 2A**), 30 out of 289 patients achieved PR (0.104, 95%CI: 0.074-0.145) (**Figure 2B**), 55 out of 289 patients achieved SD (0.190, 95%CI: 0.149-0.240) (**Figure 2C**), and 158 out of 289 patients achieved PD (0.541, 95%CI: 0.421-0.661) (**Figure 2D**).

#### 3.3.2 The ratio of ORR to DCR (%)

**Figure 3:**
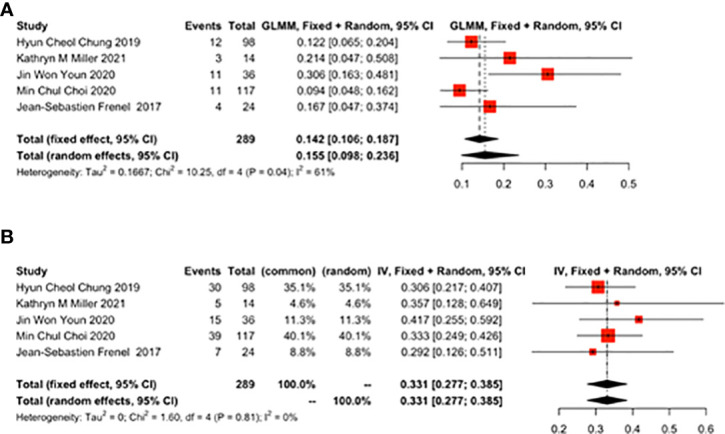
Overall results of objective response rate (ORR) and disease control rate (DCR) for patients with cervical cancer receiving pembrolizumab therapy. **(A)** ORR, **(B)** DCR.

Five studies reported the ratio of ORR to DCR. ORR was 0.155 (95% CI: 0.098-0.236) (**Figure 3A**) and DCR was 0.331 (95% CI: 0.277-0.385) (**Figure 3B**).

#### 3.3.3 OS and PFS

**Figure 4:**
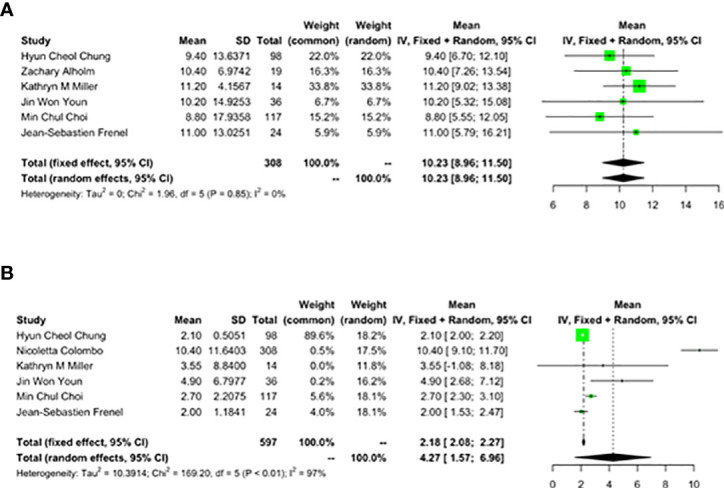
Overall survival (OS) and progression-free survival (PFS) for patients with cervical cancer receiving pembrolizumab therapy. **(A)** OS, **(B)** PFS.

Six studies reported the effect of pembrolizumab on OS and PFS in cervical cancer patients. OS was 10.23 months (95% CI: 8.96-11.50) (**Figure 4A**) and PFS was 4.27 months (95% CI: 1.57-6.96) (**Figure 4B**). There was a significant heterogeneity in the overall results of PFS (I^2^ = 97%, p<0.01) (**Figure 4B**). However, heterogeneity was not observed in OS (I^2^ = 0%, p=0.85) (**Figure 4A**).

#### 3.3.4 TTR and Death

**Figure 5:**
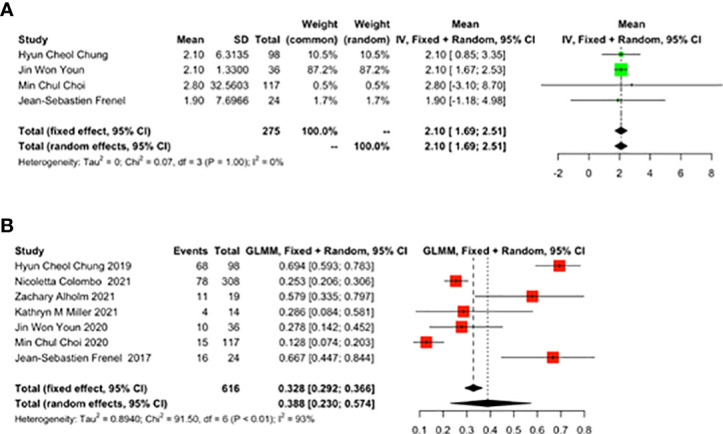
The best time to response (TTR) and 1-year mortality rate for patients with cervical cancer receiving pembrolizumab therapy. **(A)** TTR, **(B)** Death.

As reported by 4 studies, TTR was 2.10 months (95% CI: 1.69-2.51) (**Figure 5A**). As reported by 7 studies, the 1-year mortality rate was 0.388 (95% CI: 0.230-0.574) (**Figure 5B**) in cervical cancer patients treated with pembrolizumab.

#### 3.3.5 Adverse events AE

**Figure 6:**
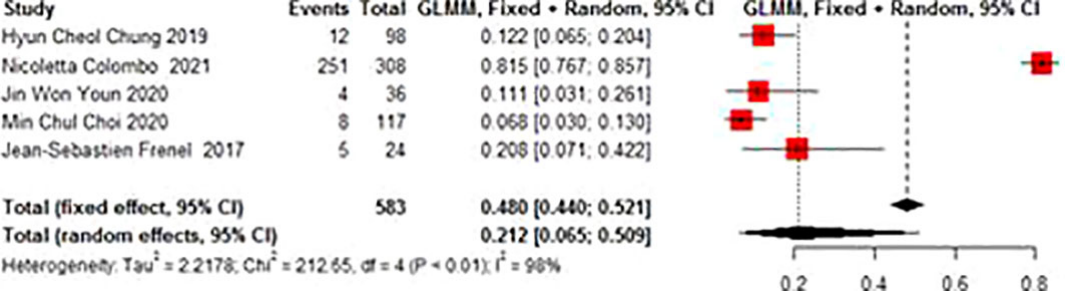
Adverse events (AE) for patients with cervical cancer receiving pembrolizumab therapy.

Adverse events mainly included abnormal liver function, hypothyroidism, neutropenia, anemia, decreased appetite, fatigue, fever, etc. The total incidence of the adverse events of grade 3 and above was 0.212 (95% CI 0.065-0.509). There was no significant heterogeneity in AE (I^2 =^ 98%, p<0.01) (**Figure 6**).

## 4 Discussion

The overall quality of the 7 studies included in this meta-analysis was high, indicating that a relatively objective result could be obtained by our study. A total of 727 cervical cancer patients received pembrolizumab in this meta-analysis. Among 727 patients, Squamous (604 cases, 83.08%), Adenocarcinoma (93 cases, 12.79%), Adenosquamous (21 cases, 2.89%) and other types (9 cases, 1.24%) were the main pathological types. Among them, Squamous accounts for more than 70% of all studies. The specific distribution is shown in the following **Table 2 cont**. As demonstrated by this single-arm meta-analysis, the complete response rate of this treatment was 2.7%, the partial response rate was 10.4%, the rate of stable disease was 19%, the disease progression rate was 54.1%, the objective response rate was 15.5%, the disease control rate was 33.1%, the median overall survival was about 10.23 months, the median progression-free survival was 4.27 months, the best response time was 2.1 months, and one-year death rate was 38.8%. Major adverse events included tolerable hepatic dysfunction, hypothyroidism, neutropenia, decreased appetite and fatigue, and the total incidence of adverse events of grade 3 and above was 21.2%.

**Table 2:** Cont. Proportion of case types included in the literature N (%).

First Author	Year	Squamous	Adenocarcinoma	Adenosquamous	other
Hyun Cheol Chung	2019	92 (93.88)	5 (5.10)	1 (1.02)	0
Nicoletta Colombo	2021	235 (76.3)	56 (18.18)	15 (4.87)	2 (0.65)
Zachary Alholm	2021	127 (97.69)	3 (2.31)	0	0
Kathryn M Miller	2021	11 (78.57)	1 (7.14)	1 (7.14)	1 (7.14)
Jin Won Youn	2020	28 (77.78)	8 (22.22)	0	0
Min Chul Choi	2020	88 (75.21)	19 (16.24)	4 (3.42)	6 (5.13)
Jean-Sebastien Frenel	2017	23 (95.83)	1 (4.17)	0	0
Overall		604 (83.08)	93 (12.79)	21 (2.89)	9 (1.24)

The 5-year survival rate of cervical cancer patients at an early stage after radical resection is about 80%. After radical radiotherapy and chemotherapy, the 5-year survival rate can still achieve about 70% even if the patients have locally advanced cervical cancer (29). However, about 28% of cervical cancer patients may have local recurrence or distant metastasis. Most cases of recurrence have been seen within 3 years, with poor prognosis, and 30-50% of the patients with locally advanced cervical cancer may relapse and eventually die of it (30, 31). Currently, the treatment approaches for recurrent/metastatic cervical cancer are limited. Treatment strategies are made primarily based on patients` physical condition, sites and ranges of recurrence/metastasis, and previous treatments they have received (32). Chemo-radiotherapy presents typically a preferred option (33, 34). Pelvic recurrence is the most common type of local recurrence, and radiotherapy or platinum-based concurrent chemoradiotherapy are standard treatments for pelvic recurrence after radical hysterectomy. Vaginal vault relapses can be treated with external radiotherapy plus brachytherapy, as compared to the nodal disease in which external radiotherapy alone is the only therapeutic option. In case when recurrent disease involves the pelvic side wall and the primary treatment included chemoradiation or surgery followed by adjuvant radiotherapy, then palliative CT is a reasonable option due to limitations of exenteration in this setting (35). However, the outcome would not be satisfactory due to complications and chronic toxic and side effects induced by radiotherapy. It has been generally accepted that cisplatin is the most effective agent for relapsed/advanced cervical cancer. Platinum-based combination regimens, such as cisplatin combined with paclitaxel, are currently recommended. For patients who had history of cis-platinum administration, carboplatin combined with paclitaxel would be the most preferred alternative, with other second-line options such as bevacizumab, docetaxel, gemcitabine, and irinotecan (36). Studies have shown that the overall survival has been improved in the cervical cancer patients who received combination treatment of bevacizumab and chemotherapy compared with those who received chemotherapy alone (37). Even so, there is no established evidence that treatment in the second-line setting improves OS compared with best supportive care. In addition, treatment of advanced cervical cancers in older patients remains controversial and poorly defined. Historically, the first reported series of patients treated at large proportion with second-line systemic treatment for recurrent or metastatic cervical cancer was performed at the Royal Marsden Hospital between 2004 and 2014. In this retrospective series, 70% of women treated with systemic therapy for recurrent or metastatic cervical cancer subsequently received second-line therapy with an ORR of 13.2%, a median PFS of 3.2 months and a median overall survival of 9.3 months (38). The only option for advanced cervical cancer patients with multiple complications is palliative care, to maintain the dignity and quality of life. Nevertheless, the treatment progress of recurrent/metastatic cervical cancer is generally slow due to the high recurrence rate after radiotherapy and chemotherapy, critical toxic and adverse events, poor tolerance, and rapid deterioration of quality of life (39).

In recent years, immunotherapy research has received increasing attention due to its sensitivity, specificity and self-renewal ability of the immune system. Good results have been achieved in the treatment of various solid tumors by immunotherapy (40). Tumor immunotherapy has also become the fourth tumor treatment strategy after surgery, chemotherapy and radiotherapy. Tumors evade the immune response by suppressing the immunosuppressive signaling pathway when the body’s immune function is at a low level. Immunotherapy aims to activate the immune system and kill tumor cells *via* the immune function in our body (41). Since the approval of ipilimumab in the United States in 2011, immune checkpoint inhibitors (ICIs) have made breakthroughs in tumor immunotherapy. Among them, the expression level of PD-L1 in cervical cancer patients is relatively high, ranging from 34.4% to 96.0% (42), which suggests that PD-1 inhibitors can be used in the treatment of cervical cancer. Therefore, ICIs are expected to be used as a potential treatment for cervical cancer. In particular, according to the KEYNOTE-158 (21) clinical trial, pembrolizumab was proved to be effective against solid tumors, including cervical cancer, and was approved by the U.S. Food and Drug Administration (FDA). Pembrolizumab is an effective humanized immunoglobulin G4 (IgG4) monoclonal antibody (mAb) with a high specificity of binding with the PD-1 receptor. Pembrolizumab can inhibit the PD-1 pathway (including PD-L1 and PD-L2) *via* dual-ligand blockade. Due to its inherent properties, pembrolizumab can inhibit tumor growth with low toxicity (43). Based on this NCCN guideline, pembrolizumab is recommended as a second-line regimen for PD-L1-positive or MSI-H/dMMR recurrent/metastatic cervical cancer. However, most of the existing studies were designed as retrospective analyses, phase I or II trials. Therefore, it is necessary to summarize the existing data to promote future research.

For recurrent/metastatic cervical cancer, cisplatin-based chemotherapy is the most common treatment (44). When using cisplatin as the only agent for chemotherapy, the median overall survival (OS) is approximately 6.5 months, the median progression-free survival (PFS) is approximately 3 months, and the remission in most patients is partial and transient (45–47). Since 1999, the American Gynecologic Oncology Group (GOG) has conducted a series of clinical trials. Among them, the GOG204 trial compared four groups of platinum-based doublet chemotherapy (including cisplatin with paclitaxel, gemcitabine, topotecan or Vinorelbine), paclitaxel combined with cisplatin was finally determined as the preferred chemotherapy regimen. The median OS was about 12.9 months, the median PFS 5.9 months, and the ORR 29.1% (48). In recent years, vascular endothelial growth factor (VEGF) has been demonstrated to promote tumor development by inducing angiogenesis, among which bevacizumab is a humanized monoclonal antibody that can specifically bind to VEGF-A (49, 50). Bradley J. Monk et al. (51) conducted a phase II trial and found that the median OS of bevacizumab was 7.29 months, the median PFS 3.4 months, and the ORR 10.9%. The results of the GOG240 study showed that the median OS of combination chemotherapy of cisplatin, paclitaxel and bevacizumab was increased to 16.8 months, and the median PFS was 8.2 months (52). Therefore, cisplatin/carboplatin-paclitaxel-bevacizumab is currently recommended as the first-line treatment in the NCCN guideline (11). Nonetheless, a significant proportion of patients died within one year after receiving systemic therapy (43%), and many patients needed second-line therapy within one year due to disease progression or toxicity (31%) (53). The results of this study showed that the median OS of pembrolizumab group was 10.23 months, the median PFS was 4.27 months, the ORR was 15.5%, and the mortality rate within one year was 38.8%, which was significantly better than that of using bevacizumab alone. The effect seemed not better compared to the first-line regimen. However, all the patients in the GOG240 study did not receive paclitaxel and platinum-based chemotherapy. In the existing studies, most patients using pembrolizumab had received at least one combination chemotherapy or radiation therapy before. According to the comparison results after disease progression, the median OS of the chemotherapy using cisplatin and paclitaxel after progression was 7.1 months, and the median OS of the chemotherapy using cisplatin + paclitaxel + bevacizumab after progression was 8.4 months (53), which were both lower than the overall survival when using pembrolizumab. At the same time, pembrolizumab in combination with standard chemotherapy has also been proposed to improve the efficacy, which is currently being explored.

In terms of safety, patients using cisplatin-based chemotherapy are prone to myelosuppression, diarrhea, and tenesmus, which may lead to treatment interruption or prolongation of treatment time. Some patients even suffer from long-term chronic diarrhea and malnutrition (54), and many patients eventually discontinue the therapy due to drug resistance (55). Bevacizumab increases the risk of specific adverse events (gastrointestinal tract perforation or fistula, thromboembolism, hypertension, etc.) especially severe adverse events, compared with chemotherapy alone (56). Current development of proteomics technology (mass spectrometry and protein array analysis) has deepened the identification of potential molecular signaling events and the proteomic characteristics of cervical and ovarian cancer, which facilitates the exploration of new therapeutic agents so as to reduce drug-resistance (57). In this meta-analysis, it was found that the incidence of the adverse events of grade 3 and above in the patients using pembrolizumab was 21.2%, mainly including tolerable liver dysfunction, hypothyroidism, neutropenia, anemia, decreased appetite, fatigue, and fever. Most of the adverse events can be improved after the therapy is discontinued. Most other PD-1/PD-L1 inhibitors, like nivolumab and atezolizumab, are still at the stage of I/II phase clinical trial so that the incidence of adverse events would be unavailable. It indicated that pembrolizumab showed significantly better safety.

On the other hand, translational research has identified a large number of potential biomarkers involved in the carcinogenesis. Within these biomarkers is included the baseline neutrophil-to-lymphocyte ratio (NLR), which is a simple haematological parameter easily obtainable in daily clinical practice. NLR has been repeatedly reported as a significant prognostic factor in advanced cancer patients. Changes in the NLR are a useful predicting factor in advanced cervical cancer patients treated with anti-PD-1/PD-L1 agents (58).

This meta-analysis has the following advantages. Firstly, this was the first article to provide evidence-based proof for the efficacy and safety of pembrolizumab in cervical cancer. Secondly, the original studies included were of high quality. At the same time, there were some limitations. First, even though we have conducted a comprehensive and systematic search in the mainstream databases, the number of the retrieved literature was still relatively small. Second, the included studies could only be used to conduct a single-arm meta-analysis, and we did not made comparison with the mainstream treatment strategies at present. Therefore, we could not directly reflect whether the treatment of pembrolizumab had advantages.

## 5 Conclusion

This single-arm meta-analysis showed that pembrolizumab could ameliorate cervical cancer to some extent and bring survival benefits. Therefore, pembrolizumab can be used as a promising treatment option for advanced/recurrent cervical cancer. However, due to the limitation of the published original literature, the existing evidenve is still not enough to support us to complete RCT-based meta-analysis. Thus, we look forward to more center RCTs which are not limited to ethnicity in the future to explore the specific advantages of pembrolizumab in cervical cancer treatment.

## Data availability statement

The raw data supporting the conclusions of this article will be made available by the authors, without undue reservation.

## Author contributions

LQ and JC contributed the central idea, analysed most of the data and wrote the main manuscript text. NL, AL and XW contributed to refining the ideas, collecting the data, carrying out additional analyses and revising the manuscript. All authors have read and approved the final version of the manuscript. All authors contributed to the article and approved the submitted version.

## Conflict of interest

The authors declare that the research was conducted in the absence of any commercial or financial relationships that could be construed as a potential conflict of interest.

## Publisher’s note

All claims expressed in this article are solely those of the authors and do not necessarily represent those of their affiliated organizations, or those of the publisher, the editors and the reviewers. Any product that may be evaluated in this article, or claim that may be made by its manufacturer, is not guaranteed or endorsed by the publisher.
